# Lychee Peel Extract Ameliorates Hyperuricemia by Regulating Uric Acid Production and Excretion in Mice

**DOI:** 10.3390/cimb47020076

**Published:** 2025-01-25

**Authors:** Zhenwang Guo, Li Zhang, Jinlei Liu, Ziming Yang

**Affiliations:** 1 Guangxi Key Laboratory of Plant Functional Phytochemicals and Sustainable Utilization, Guangxi Institute of Botany, Guangxi Zhuang Autonomous Region and Chinese Academy of Sciences, Guilin 541006, China; gzhw2005214@163.com (Z.G.); zl@gxib.cn (L.Z.); ljl@gxib.cn (J.L.); 2 Guangxi School of Chinese Medicine, Nanning 530001, China

**Keywords:** lychee peel, hyperuricemia, polyphenolic compounds, XOD, oxidative stress

## Abstract

Lychee peel generated during the industrial processing of lychee fruit are currently disposed of as agricultural waste. This study investigates the primary components of lychee peel extract (LPE) and the regulatory mechanisms of LPE on reducing uric acid (UA). Mice were injected with hypoxanthine and potassium oxonate to induce hyperuricemia and concurrently orally administered LPE. The analysis of the LPE composition reveals a predominance of polyphenolic compounds, including (-)-epicatechin, (-)-epigallocatechin, and procyanidin A2. In vitro tests have demonstrated that the LPE significantly inhibits the activity of xanthine oxidase (XOD). In vivo studies showed that LPE can reduce UA levels in hyperuricemia mice. Further mechanistic insights indicate that LPE inhibits hepatic XOD activity, thereby reducing UA synthesis within the organism. It also decreases the protein expression of urate transporter 1 (URAT1) and glucose transporter 9 (GLUT9), which leads to diminished UA reabsorption and increased excretion of UA. Additionally, LPE enhances the activity of superoxide dismutase (SOD) while simultaneously reducing malondialdehyde (MDA) contents, thereby improving antioxidant capacity in mice. Our findings indicate that LPE not only inhibits the production of UA but also promotes its elimination, positioning it as a promising candidate for UA-lowering agents.

## 1. Introduction

China is the leading producer of lychee (*Litchi chinensis* Sonn.), accounting for more than half of the worldwide production [1]. The industrial processing of lychee produces a significant amount of agricultural waste, primarily consisting of the core and peel. This agricultural waste is often incinerated or disposed of in landfills, thereby exacerbating environmental pressures. Therefore, efforts should be directed towards transforming the agricultural crop waste generated during production and processing into valuable resources and products that can benefit humanity. A promising application of agricultural waste entails the extraction of residual biomass for the purpose of obtaining plant-derived compounds. Plant tissues are widely recognized for their rich and diverse array of biologically active compounds [2]. Consequently, they hold significant potential as source materials for the extraction of valuable phytochemicals. Lychee peels have indeed been recognized as potential sources of beneficial phytochemicals. Literature reviews and our research have shown that lychee peel is rich in polyphenolic compounds, featuring substances such as (-)-epicatechin, (+)-catechin, quercetin, procyanidin A2, procyanidin B2, rutin, and (-)-epigallocatechin gallate, with (-)-epicatechin being predominant [3,4,5,6]. These phytochemicals exhibit a diverse range of protective functions, including anti-inflammatory, antioxidant, anti-tumor, anti-atherosclerotic, lipid-lowering, and hypo-glycemic properties [4,6,7,8,9,10].

Hyperuricemia is a widespread metabolic disorder observed globally, with a higher prevalence in males compared to females [11]. Although the incidence of hyperuricemia is increasing, the management of gout remains inadequate in numerous countries, with only 33% to 50% of affected individuals receiving treatments that lower uric acid (UA) levels. Traditionally considered a primary cause of gout and gouty arthritis, recent research has expanded our understanding of hyperuricemia. The evidence suggests that elevated levels of UA can worsen the progression of chronic kidney disease and cardiovascular disorders by initiating inflammatory responses and stimulating the renin–angiotensin system [12,13].

Elevated UA levels in humans stem from three primary causes: (1) A high dietary intake of purines, such as animal offal, seafood, and red meats, can lead to an increase in metabolic by-products. When excessive purines are not promptly excreted from the body, they can elevate blood UA levels [14]. (2) Disorders in purine metabolism, specifically anomalies in the enzymes responsible for processing purines, can lead to increased production of UA. Unlike other animals, humans lack the enzyme uricase, which is responsible for breaking down UA into allantoin for excretion. As a result, purines are exclusively eliminated as UA in humans. Xanthine oxidase (XOD) assumes a crucial role in the transformation of purine into UA, thereby making it a prevalent target in the research of lowering UA [15,16]. (3) UA excretion that is impaired; UA is predominantly excreted via renal filtration, undergoing bidirectional transport processes in renal tubules, which encompass the reabsorption, secretion, and post-secretion reabsorption phases. This transport is facilitated by specific UA transporters, with urate transporter 1 (URAT1) and glucose transporter 9 (GLUT9) primarily mediating reabsorption and organic anion transporter 1 (OAT1) and organic anion transporter 3 (OAT3) overseeing secretion. Dysfunctions in these transporters can diminish UA excretion, contributing to hyperuricemia [17,18,19]. Current therapeutic strategies for hyperuricemia focus on reducing UA synthesis and enhancing its excretion. Allopurinol, an XOD inhibitor, prevents the conversion of xanthine and hypoxanthine to UA and is widely prescribed despite potential adverse effects like skin rashes and kidney damage. According to international guidelines, allopurinol is recommended for patients with gout or kidney stones, with febuxostat suggested for those with renal issues due to its superior efficacy and lower risk of liver or kidney damage, albeit at a higher cost [20,21,22]. Additionally, enhancing UA excretion is crucial. Agents such as probenecid and benzbromarone, which block UA reabsorption in the renal tubules, effectively lower UA levels. Another approach involves administering uricase intravenously to break down UA, utilizing drugs like pegloticase and rasburicase, which are particularly useful given the absence of natural uricase activity in humans [23,24].

As socio-economic conditions evolve and lifestyles change, the prevalence of hyperuricemia is anticipated to rise significantly, posing substantial burdens on society, families, and individuals. Given these implications, the need for effective UA-lowering strategies is critical. Due to their low toxicity and a reduced likelihood of drug resistance, natural products are increasingly being recognized for their value in the healthcare sector. Considering that lychee peel biowaste may have significant economic potential, several studies have examined its possible use as a source of pharmacological [6,7,8,9]. Despite the extensive documentation of the protective functions attributed to lychee peel, its effects on UA levels remain unexplored. The peel of lychee is rich in polyphenols, which presents a novel perspective for the treatment of hyperuricemia due to their multi-component, multi-target, and highly active characteristics. In this study, lychee peel extract (LPE) was derived from the peels of lychee fruit, and its inhibitory effect on XOD activity was assessed in vitro. A combination of potassium oxazinate and hypoxanthine was utilized to establish an animal model of hyperuricemia. The UA-lowering effect and mechanism of LPE were investigated by assessing relevant biochemical indexes in urine, blood, liver, and kidney, as well as examining the expression of related proteins in the kidney.

## 2. Materials and Methods

### 2.1. Materials and Methods

Antibodies against glyceraldehyde-3-phosphate dehydrogenase (GAPDH, 20220603), URAT1 (20230315), and GLUT9 (20221220) were purchased from Cell Signaling Technology (Beverly, MA, USA). The gallic acid (Yz011623), (-)-epicatechin (Yz112421), procyanidin A2 (Yz0804222), rutin (Yz111224), (-)-epigallocatechin (Yz020726), (+)-catechin (Yz020723), procyanidin B1 (Yz040221), procyanidin B2 (Yz113023), protocatechuic acid (Yz040229), and vanillic acid (Yz040521) standards were purchased from Plant Origin Biological (Nanjing, China). Folin–Ciocalteu reagent (ST2070) was purchased from Beyotime (Shanghai, China). XOD (E0083) was purchased from Njduly (Nanjing, China). In Lingshan County, Guangxi, an agricultural ecological park was used to gather the lychee fruit. We used 8-week-old, specific-pathogen-free (SPF) C57BL/6 mice certified by SCXK (Xiang) 2020-0004 from Hunan SJA Laboratory Anima Co., Ltd. (Changsha, China). This company also supplied the standard mouse diet, authorized under license numbered SCXK (Xiang) 2020-0002.

### 2.2. Preparation of LPE

The lychee peel (thin white semitransparent layer and hard rough coat) extraction process utilized our previously established method [6]. Fresh peels were initially steeped in 80% ethanol (10% *w*/*v*) for a week and subsequently filtered. The filtrate was centrifuged at 3500 rpm for 10 min, collecting the supernatant. The mixture was subjected to column chromatographic purification using an XDA-7 macroporous resin column, followed by elution with a 60% ethanol aqueous solution. A rotary evaporator N-3100 (Eyela, Tokyo, Japan) was utilized to concentrate the eluate under a vacuum at 95 kPa, with a heating bath temperature of 40 °C and a cooling water temperature of 15 °C. Subsequently, the concentrate was dried using an HF-015 spray dryer (Hefan, Shanghai, China) to produce the LPE.

### 2.3. Determination of Total Polyphenol Content of LPE

The Folin–Ciocalteu technique was used to determine the total phenolic content [6]. The gallic acid standard solution was made up at a concentration of 0.1 mg/mL. A total of 0.5 mL of Folin–Ciocalteu reagent and 1.5 mL of 15% Na2CO3 solution were sequentially added to 10 mL test tubes containing 0.1, 0.2, 0.3, 0.4, 0.5, 0.6, and 0.7 mL of the gallic acid standard solution. The mixture was then adjusted to a volume of 10 mL with distilled water, thoroughly mixed, and subsequently heated in a water bath at 75 °C for 15 min. At 760 nm, the absorbance was recorded. A calibration curve was plotted correlating gallic acid concentrations with absorbance readings via linear regression. A similar procedure was applied to a 0.1 mg/mL LPE solution, and the phenolic content was calculated from the regression equation, ensuring accuracy through triplicate measurements.

### 2.4. Determination of Individual Components in LPE

The preparation of standard solutions (2 mg/mL) of (-)-epicatechin, procyanidin A2, rutin, (-)-epigallocatechin, (+)-catechin, procyanidin B1, procyanidin B2, protocatechuic acid, and vanillic acid in methanol was conducted. Each sample was passed through a membrane filter with a pore size of 0.22 μm. The standard substance content was quantified using a high-performance liquid chromatograph (HPLC) (Agilent, Palo Alto, CA, USA) and compared against the standard curve. The operating settings were a temperature of 35 °C, a flow rate of 0.8 mL/min, and a Shimadzu Inertsil ODS-3 C18 column with dimensions 250 mm × 4.6 mm and a particle size of 5 μm. A value of 250 nm was chosen as the detecting wavelengths. The mobile phases were 0.1% formic acid in water (Phase A) and acetonitrile (Phase B), and they were eluted using a gradient method. The concentration of each compound in the LPE was calculated by integrating the peak areas from the HPLC chromatograms into the constructed standard curves, following the external standard method.

### 2.5. The Effect of LPE on the Activity of XOD

XOD catalyzes the oxidation of hypoxanthine into UA, which exhibits a distinct absorption peak at 295 nm. To evaluate the influence of LPE on XOD activity, changes in the optical density (OD) value at 295 nm are monitored minutely. The reaction steps are as follows: A total of 100 μL of various concentrations of LPE and allopurinol (used as a positive control) were added to the wells of a 96-well plate. Subsequently, 50 μL of XOD solution at 0.1 U/L was added, followed by incubation at 37 °C for 5 min. Then, a 0.05 mmol/L hypoxanthine solution was introduced to start the enzymatic reaction for a duration of 5 min. The inhibition percentage of XOD by the test sample was determined using a specific formula:

XOD inhibition rate (%) = (Ac − As)/Ac × 100%

Here, Ac is the reaction rate of the control group, and As represents the reaction rate of the sample group.

### 2.6. Effect of LPE on Hyperuricemia Mice

#### 2.6.1. Experimental Design and Administration

The Research Ethics Committee of the Guangxi Institute of Botany, Guangxi Zhuang Autonomous Region and the Chinese Academy of Science, granted approval for the animal experiment procedures (approval ID: GXZW-2023031005). Once the mice had adjusted to their new environment for 7 days, they were divided into six groups based on their weight: the blank control group (BCG), the model control group (MCG), the low dosage group (LDG), the medium dose group (MDG), the high dose group (HDG), and the positive control group (PCG). Each group consisted of 12 mice. The oral administration of LPE was performed at doses of 100, 200, and 400 mg/kg for the LDG, MDG, and HDG, respectively. While the BCG and MCG receive distilled water in volumes proportional to their weight, the PCG receives 5 mg/kg of allopurinol. The treatment lasts for 14 days and is administered every day.

#### 2.6.2. Treatment of Experimental Animals

On the seventh day, 2 h after administration, all mice except for the BCG mice were subjected to a dual administration procedure: an intraperitoneal injection of 300 mg/kg hypoxanthine and a subcutaneous injection of 100 mg/kg potassium oxonate. Urine was collected a 24 h period in metabolic cages, measured volumetrically using a graduated cylinder, and analyzed for UA content via a standard reagent kit (Jiancheng, Nanjing, China). On the 14th day of the experiment, following a period of fasting, the mice were weighed using an electronic scale. Two hours prior to euthanasia, a repeat dose of hypoxanthine and potassium oxonate was administered, as had been performed on the seventh day. Following this, the mice were given pentobarbital sodium to make them unconscious. Blood samples were taken and spun in a centrifuge, and the serum was kept at 4 °C for further biochemical testing. Following the euthanasia procedure, the following organ indices were determined by removing and weighing the liver and kidney tissues:

Liver index = (liver weight × 1000)/mouse weight

Kidney index = (kidney weight × 1000)/mouse weight

Tissue samples from consistent regions of each liver and kidney were homogenized in saline for further biochemical testing. Tissue samples from consistent regions of each kidney were preserved in cryogenic tubes and stored in liquid nitrogen for subsequent Western blot analysis.

#### 2.6.3. Measurement of Biochemical Indicators in Mice

Malondialdehyde (MDA) content and superoxide dismutase (SOD) activity in the serum, liver, and kidney were determined using previously established methods [25]. The serum levels of UA, alanine transaminase (ALT), aspartate transaminase (AST), blood urea nitrogen (BUN), and creatinine (Cre) were measured utilizing commercial kits (Jiancheng, Nanjing, China). Additionally, hepatic XOD activity was quantified using specific assay kits provided by the same supplier.

#### 2.6.4. Extraction of Total Protein from Kidney Tissue

The frozen kidney tissues were retrieved from liquid nitrogen storage. For protein extraction, approximately 100 mg of tissue was sectioned, homogenized in 400 μL of RIPA lysis buffer with an ultrasonic crusher, and centrifuged at 12,000× *g* for 15 min at 4 °C. The resulting supernatant, which contains the total protein of the kidney, was preserved for subsequent analysis.

#### 2.6.5. Western Blot

An assay kit (Beyotime, Shanghai, China) was used to measure the protein concentrations in the samples. After combining the sample protein supernatant with a loading buffer, it was boiled for 5 min to denature it. Finally, the mixture was subjected to SDS-PAGE for analysis. Based on the molecular weight of the protein to be separated, a separation glue of the required concentration is prepared. Each sample containing 30 μg of protein was loaded onto a gel for separation. Protein bands were identified using enhanced chemiluminescence and semi-quantitatively assessed using an image processing system. The gray values were utilized to indicate the levels of protein.

### 2.7. Statistical Analysis

The experimental data were expressed as mean ± standard deviation. The data were analyzed statistically with the help of GraphPad Prism 8 (GraphPad, San Diego, CA, USA). To examine the differences between the groups, one-way ANOVA was used, and for multiple comparisons, Tukey’s post hoc test was employed. Statistical significance was determined when the *p* < 0.05.

## 3. Results and Discussion

### 3.1. Composition of LPE

Table 1 indicates that the LPE contains 51.70% total polyphenols, with its main components being (-)-epicatechin, (-)-epigallocatechin, procyanidin A2, procyanidin B1, procyanidin B2, and (+)-catechin. Notably, (-)-epicatechin was the most abundant, comprising 10.30% of the extract, aligning with findings from previous studies [6].

### 3.2. In Vitro Inhibitory Effect of LPE on XOD

**Figure 1:**
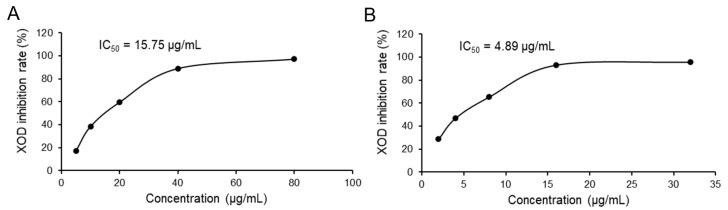
XOD inhibition by LPE and allopurinol. (**A**) Effect of LPE on XOD activity. (**B**) Effect of allopurinol on XOD activity.

Figure 1 depicts the dose-dependent inhibitory action of LPE and allopurinol on XOD. The LPE progressively inhibited XOD activity with increasing concentrations, demonstrating an IC50 value of 15.75 μg/mL. In comparison, allopurinol exhibited a similar pattern of inhibition, with an IC50 value of 4.89 μg/mL, indicating potent efficacy higher than that of the LPE. These findings highlight that LPE exhibits a notable capacity to inhibit XOD in vitro. Although LPE exhibits a lower potency compared to the pharmaceutical standard, its inhibitory efficacy against XOD is considered promising as a natural product. This potential underscores the appropriateness of LPE as a candidate for further development. As previously outlined, XOD plays a crucial role in catalyzing the conversion of purines to UA [3]. This positions the XOD inhibitory capabilities of LPE as a strategic avenue to mitigate UA synthesis. Further research will extend these findings by assessing the impact of LPE on UA metabolism in hyperuricemia mice.

### 3.3. Effects of LPE on UA Content and XOD Activity in Hyperuricemia Mice

**Figure 2:**
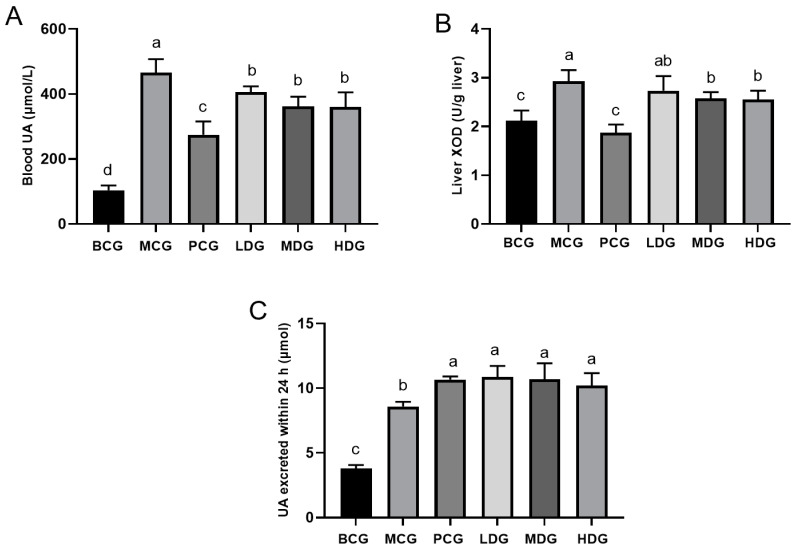
Effect of LPE on UA content and XOD activity in hyperuricemia mice. (**A**) UA content in mouse serum. (**B**) XOD activity in mouse liver. (**C**) UA excretion in mouse over a 24 h period. Values are expressed as mean ± standard deviation (*n* = 12 in each group). The values with different letters (a, b, c or d) are significantly different (*p* < 0.05) between each group.

Numerous methods exist for inducing hyperuricemia in animal models [26]; they are generally categorized as follows: (1) supplementation with UA or its precursors, like hypoxanthine; (2) inhibition of uricase, using agents such as potassium oxonate; and (3) reduction in renal UA excretion through substances like ethambutol. The development of an effective animal model is essential for evaluating the therapeutic efficacy of drugs. In this study, a combination of intraperitoneal hypoxanthine and subcutaneous potassium oxonate injections was utilized to successfully establish a hyperuricemia model, as shown in Figure 2. This method aligns with previous studies that have similarly employed these compounds to induce hyperuricemia [27]. As depicted in Figure 2A, compared to the MCG mice, our analysis showed that all mice treated with LPE had significantly lower UA levels (*p* < 0.05). The UA levels in the LDG mice dropped by 12.86%; in the MDG mice, by 22.53%; and in the HDG mice, by 22.75%. Furthermore, XOD activity was significantly higher in the MCG mice compared to the BCG mice. However, it was notably reduced in both the medium and high-dose LPE groups compared to the MCG (*p* < 0.05), as depicted in Figure 2B. The observed increase in XOD activity among the hyperuricemia mice likely resulted from the elevated levels of hypoxanthine in the body, thereby inducing XOD activity and speeding up hypoxanthine metabolism. These findings confirm the potential of LPE to reduce XOD activity, indicating its function in inhibiting UA production. This is further supported by previous in vitro studies demonstrating the extract’s inhibitory effect on XOD. As shown in Figure 2C, relative to the BCG mice, there was a notable increase in the 24 h UA excretion observed in the MCG mice, achieving statistical significance (*p* < 0.05). Similarly, the allopurinol-treated mice exhibited a significant enhancement in UA excretion over the same period when compared to the MCG mice (*p* < 0.05). This aligns with existing research, indicating that allopurinol not only suppresses UA synthesis but also facilitates its excretion [28]. Furthermore, all three dosage groups treated with LPE demonstrated a significant elevation in 24 h UA excretion compared to the MCG. This suggests that LPE effectively reduces UA levels by both inhibiting its production and enhancing its excretion.

### 3.4. Effects of LPE on Kidney Function in Mice with Hyperuricemia

**Figure 3:**
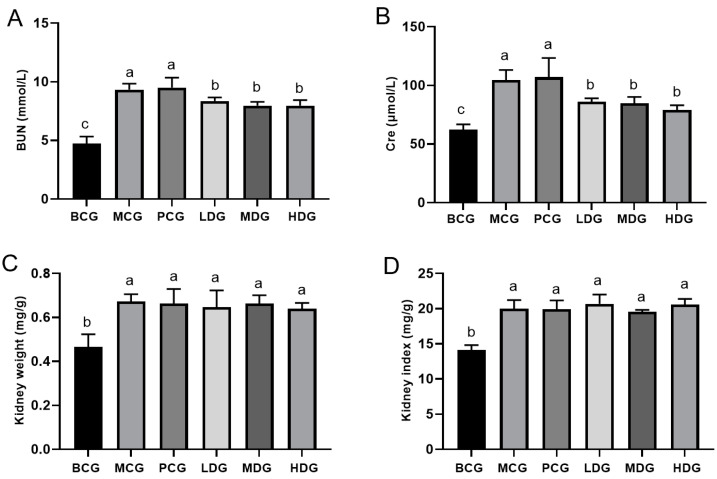
Effect of LPE on renal function in hyperuricemia mice. (**A**) BUN levels in mouse serum. (**B**) Cre levels in mouse serum. (**C**) Kidney weight. (**D**) Kidney index. Values are expressed as mean ± standard deviation (*n* = 12 in each group). The values with different letters (a, b or c) are significantly different (*p* < 0.05) between each group.

The kidney index serves as a crucial metric for evaluating renal health, where an increase may signal potential renal pathology or dysfunction. Nonetheless, given its dependency on weight, the kidney index can be subject to biases, necessitating the consideration of both kidney weight and index for more comprehensive assessments. Cre and BUN are established biochemical markers for renal function evaluation. Elevated Cre and BUN levels typically signify renal impairment [29]. As depicted in Figure 3, the MCG mice exhibited significantly higher BUN and Cre levels, alongside increased kidney weight and index compared to the BCG mice (*p* < 0.05), confirming the deleterious effects on renal function of the combined administration of potassium oxonate and hypoxanthine, in line with prior studies [30]. The allopurinol-treated mice showed no notable differences in BUN, Cre, kidney weight, and kidney index relative to the MCG mice (*p* > 0.05), suggesting that while allopurinol is effective in reducing UA levels, it does not ameliorate renal damage induced by the treatment with potassium oxonate and hypoxanthine. In contrast, the treatment with LPE at various doses significantly reduced both BUN and Cre levels compared to the MCG mice (*p* < 0.05). However, the changes in kidney weight and index were not statistically significant (*p* > 0.05). These findings indicate that LPE may have a protective effect against renal damage caused by potassium oxonate and hypoxanthine. Nevertheless, it is essential to conduct further verification through renal toxicological studies.

### 3.5. Effects of LPE on Liver Function in Hyperuricemia Mice

**Figure 4:**
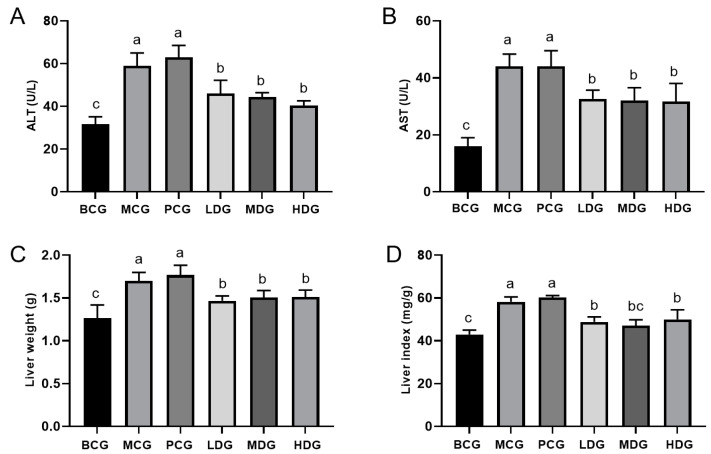
Effect of LPE on liver function in hyperuricemia mice. (**A**) ALT activity in mouse serum. (**B**) AST activity in mouse serum. (**C**) Liver weight. (**D**) Liver index. Values are expressed as mean ± standard deviation (*n* = 12 in each group). The values with different letters (a, b, or c) are significantly different (*p* < 0.05) between each group.

The liver index is crucial for toxicological assessments as it reflects the potential harm caused by drugs. However, it is important to note that this index may be influenced by various factors. Therefore, it is necessary to include additional biochemical indexes related to liver function in order to conduct a comprehensive analysis. ALT and AST are commonly used as standard biomarkers to assess liver health. Normally, these enzymes are found in the bloodstream at low levels; however, elevated levels may indicate damage or necrosis of hepatic cells [31]. Based on the data presented in Figure 4, it is clear that the MCG mice exhibited significant increases in ALT and AST levels, as well as liver weight and liver index compared to the BCG mice (*p* < 0.05). This confirms the presence of liver impairment in the mice treated with potassium oxonate and hypoxanthine. The mice treated with allopurinol did not exhibit significant differences in ALT, AST, liver weight, or liver index compared to the MCG mice (*p* > 0.05). This suggests that while allopurinol effectively reduces UA levels, it does not alleviate liver damage induced by potassium oxonate and hypoxanthine. Conversely, the groups treated with LPE at three varying doses showed notably lower levels of ALT and AST, alongside reductions in liver weight and index (*p* < 0.05). These observations suggest that LPE may confer protective benefits against hepatic damage triggered by potassium oxonate and hypoxanthine. Nevertheless, it is essential to conduct further verification through liver toxicological studies. The observed protective effect is consistent with previous findings that have demonstrated the hepatoprotective properties of LPE in db/db diabetic mice [6].

### 3.6. Effect of LPE on Antioxidant Capacity in Hyperuricemia Mice

**Figure 5:**
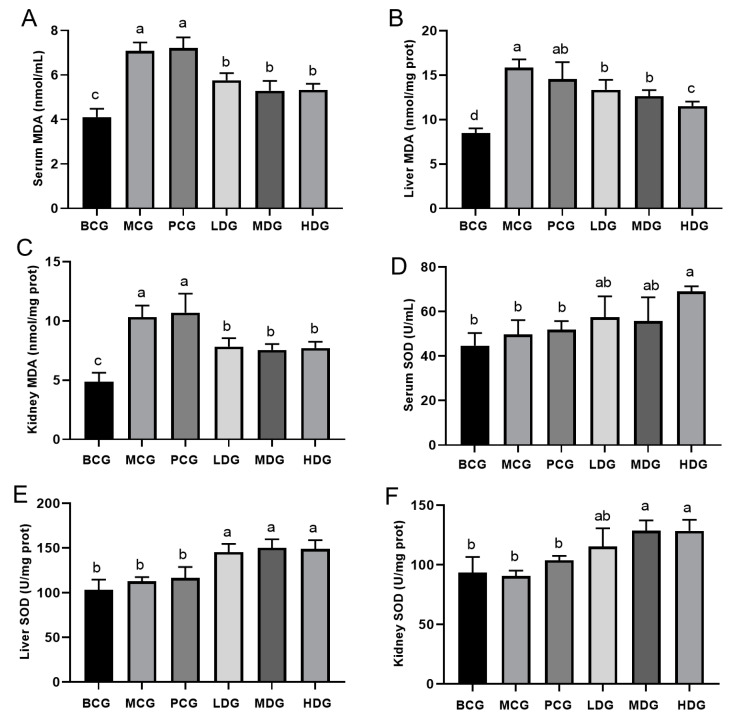
Effect of LPE on antioxidant capacity in hyperuricemia mice. (**A**) Serum MDA content. (**B**) Liver MDA content. (**C**) Kidney MDA content. (**D**) Serum SOD activity. (**E**) Liver SOD activity. (**F**) Kidney SOD activity. Values are expressed as mean ± standard deviation (*n* = 12 in each group). The values with different letters (a, b, c, or d) are significantly different (*p* < 0.05) between each group.

Oxidative stress is linked to a variety of diseases, and hyperuricemia has been reported to cause oxidative imbalances [32]. Previous research has demonstrated that extracts derived from lychee peel exhibit superior antioxidant capacity [2,6,33]. MDA is the final product of lipid peroxidation and is frequently utilized as a biomarker to assess the degree of oxidative stress [34]. Figure 5A–C demonstrate that the MDA levels in the blood, liver, and kidney of mice in the MCG were significantly elevated compared to those observed in the BCG. This finding substantiates the occurrence of oxidative stress in mice subjected to treatment with potassium oxonate and hypoxanthine. The hyperuricemia mice treated with allopurinol did not exhibit a reduction in MDA levels in the blood, liver, and kidney. This finding suggests that while allopurinol effectively lowers UA levels, it does not mitigate oxidative stress. Conversely, the levels of MDA in the groups treated with LPE were significantly reduced. This suggests that the oxidative stress induced by potassium oxonate and hypoxanthine can be effectively mitigated by LPE. SOD is a crucial endogenous antioxidant that effectively eliminates oxygen free radicals, thereby preventing their accumulation and the subsequent oxidative damage to cells [35]. The results illustrated in Figure 5D–F indicate that the blood, liver, and kidney of the MCG mice did not show significant differences in SOD activity when compared to the BCG mice. The hyperuricemia mice treated with allopurinol did not exhibit an enhancement in SOD activity in the blood, liver, or kidney. Conversely, in the LPE-treated group, the activity of SOD in the blood, liver, and kidneys of mice was significantly elevated compared to that observed in the MCG mice. Figure 5 indicates that LPE may enhance antioxidant enzyme activity and mitigate oxidative stress. Collectively, these findings indicate that LPE exhibits a strong antioxidant capacity in hyperuricemia mice. Furthermore, the UA-lowering effect of LPE may be associated with this property.

### 3.7. Impact of LPE on URAT1 and GLUT9 Protein Expression in Renal Tissues of Hyperuricemia Mice

**Figure 6:**
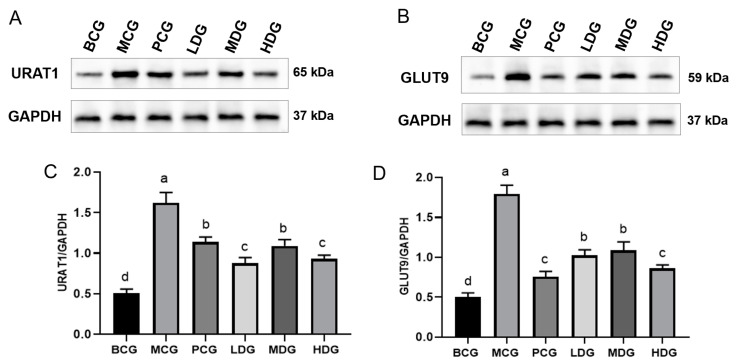
Effects of LPE on the protein expression of URAT1 and GLUT9 in the kidney of mice. (**A**) URAT1 protein expression. (**B**) GLUT9 protein expression. (**C**) Quantification of URAT1 protein levels. (**D**) Quantification of GLUT9 protein levels. Values are expressed as mean ± standard deviation (*n* = 3 in each group). The values with different letters (a, b, c, or d) are significantly different (*p* < 0.05) between each group.

More than 90% of hyperuricemia is attributed to decreased excretion of UA, with the kidneys playing a crucial role in this process by eliminating approximately 70% of UA, while the remaining amount is expelled through the intestines [36]. UA excretion is mediated by specific transport proteins within the renal tubular epithelial cells, notably URAT1 and GLUT9, which are primarily responsible for the reabsorption of UA [37]. Research indicates that the suppression or genetic deletion of URAT1 and GLUT9 can markedly enhance UA elimination [38,39,40,41], positioning these genes as significant targets for therapeutic interventions aimed at lowering UA. Figure 6 illustrates that the expression levels of URAT1 and GLUT9 were notably higher in the MCG mice compared to the BCG mice (*p* < 0.05), indicating that the administration of potassium oxonate and hypoxanthine promotes renal UA reabsorption in mice. Conversely, the allopurinol-treated mice displayed a notable decrease in the expression of these proteins compared to the MCG mice (*p* < 0.05), aligning with the enhanced UA excretion effects observed, as discussed in Section 3.3. Similarly, the treatment with LPE led to a significant reduction in URAT1 and GLUT9 levels in comparison to the MCG mice (*p* < 0.05), indicating a reduction in renal UA reabsorption and an increase in excretion, which corroborates the observations made in Section 3.3 regarding the extract’s efficacy in promoting UA excretion.

## 4. Conclusions

This study successfully extracted polyphenolic compounds from lychee peel and measured the contents of primary components such as (-)-epicatechin, (-)-epigallocatechin, procyanidin A2, procyanidin B1, procyanidin B2, and (+)-catechin. The predominant compound identified in the extract was (-)-epicatechin, comprising 10% of the total content. In vitro assessments revealed that the LPE significantly inhibits XOD, indicating its capability to decrease UA synthesis. A hyperuricemia model was established in mice using potassium oxonate and hypoxanthine, where it was observed that the LPE not only reduced serum UA levels but also enhanced 24 h UA excretion and mitigated liver and kidney damage caused by potassium oxonate and hypoxanthine. Further analysis has indicated that the effects of LPE on reducing UA levels are likely due to the inhibition of hepatic XOD activity, the downregulation of renal URAT1 and GLUT9 expression, and the restoration of oxidative balance within the body. Our findings indicate the potential utility of LPE as a functional supplement for mice with hyperuricemia. However, there are fundamental differences between the physiological systems of animals and humans. Furthermore, the hyperuricemia animal disease models utilized in research may not accurately reflect the actual conditions experienced by humans, thereby limiting the applicability of animal testing in clinical contexts. Therefore, further research is necessary to explore the possible beneficial effects of LPE in humans suffering from hyperuricemia. Due to the potential health risks associated with certain UA-lowering medications, there is a growing interest in exploring natural plant-derived alternatives for UA reduction. In this context, lychee peel waste emerges as one of the most promising sources of natural UA-lowering agents, primarily owing to its rich composition of biologically active compounds and the cost-effectiveness of the raw material. Collectively, these results suggest that LPE acts by both inhibiting UA production and enhancing its excretion, supporting its potential utility as a functional food or dietary supplement for the management of hyperuricemia.

## Figures and Tables

**Table 1:** Bioactive components of LPE.

Item	LPE
Total polyphenols (‰)	517.04 ± 18.12
(-)-Epicatechin (‰)	102.96 ± 2.49
(-)-Epigallocatechin (‰)	31.12 ± 0.25
Procyanidin A2 (‰)	16.92 ± 0.21
Procyanidin B1 (‰)	13.57 ± 0.58
Procyanidin B2 (‰)	5.75 ± 0.08
(+)-Catechin (‰)	2.34 ± 0.26
Vanillic acid (‰)	0.28 ± 0.01
Rutin (‰)	0.21 ± 0.02
Protocatechuic acid (‰)	0.16 ± 0.01

Values are presented as the mean ± standard deviation of triplicate experiments.

## Data Availability

The original contributions presented in the study are included in the article; further inquiries can be directed to the corresponding authors.
